# Regio- and stereospecific assembly of di­spiro­[indoline-3,3′-pyrrolizine-1′,5′′-thia­zolidines] from simple precursors using a one-pot procedure: synthesis, spectroscopic and structural characterization, and a proposed mechanism of formation

**DOI:** 10.1107/S2053229620009791

**Published:** 2020-07-22

**Authors:** Pablo Romo, Jairo Quiroga, Justo Cobo, Christopher Glidewell

**Affiliations:** a Grupo de Investigación de Compuestos Heterocíclicos, Departamento de Química, Universidad del Valle, AA 25360 Cali, Colombia; b Departamento de Química Inorgánica y Orgánica, Universidad de Jaén, 23071 Jaén, Spain; c School of Chemistry, University of St Andrews, Fife KY16 9ST, Scotland

**Keywords:** heterocyclic com­pound, synthesis, indoline, pyrrolizine, dipolar cyclo­addition, di­spiro com­pound, NMR spectroscopy, crystal structure, mol­ecular conformation, hydrogen bonding, supra­molecular assembly

## Abstract

The synthesis and characterization of three new di­spiro­[indoline-3,3′-pyrrolizine-1′,5′′-thia­zolidine] com­pounds are reported, together with the crystal structures of two of them. (3*RS*,1′*SR*,2′*SR*,7a′*SR*)-2′-(4-Chloro­phen­yl)-1-hexyl-2′′-sulfanyl­idene-5′,6′,7′,7a′-tetra­hydro-2′*H*-di­spiro­[indoline-3,3′-pyrrolizine-1′,5′′-thia­zolidine]-2,4′′-dione, C_28_H_30_ClN_3_O_2_S_2_, (I), (3*RS*,1′*SR*,2′*SR*,7a′*SR*)-2′-(4-chloro­phen­yl)-1-benzyl-5-methyl-2′′-sulfanyl­idene-5′,6′,7′,7a′-tetra­hydro-2′*H*-di­spiro­[in­doline-3,3′-pyrrolizine-1′,5′′-thia­zolidine]-2,4′′-dione, C_30_H_26_ClN_3_O_2_S_2_, (II), and (3*RS*,1′*SR*,2′*SR*,7a′*SR*)-2′-(4-chloro­phen­yl)-5-fluoro-2′′-sulfanyl­idene-5′,6′,7′,7a′-tetra­hydro-2′*H*-di­spiro­[indoline-3,3′-pyrrolizine-1′,5′′-thia­zolidine]-2,4′′-dione, C_22_H_17_ClFN_3_O_2_S_2_, (III), were each isolated as a single regioisomer using a one-pot reaction involving l-proline, a substituted isatin and (*Z*)-5-(4-chloro­benzyl­idene)-2-sulfanyl­idene­thia­zolidin-4-one [5-(4-chloro­benzyl­idene)rhodanine]. The com­positions of (I)–(III) were established by elemental analysis, com­plemented by high-resolution mass spectrometry in the case of (I); their constitutions, including the definition of the regiochemistry, were established using NMR spectroscopy, and the relative configurations at the four stereogenic centres were established using single-crystal X-ray structure analysis. A possible reaction mechanism for the formation of (I)–(III) is proposed, based on the detailed stereochemistry. The mol­ecules of (I) are linked into simple chains by a single N—H⋯N hydrogen bond, those of (II) are linked into a chain of rings by a combination of N—H⋯O and C—H⋯S=C hydrogen bonds, and those of (III) are linked into sheets by a combination of N—H⋯N and N—H⋯S=C hydrogen bonds.

## Abstract 2

Di­spiro­[indoline-3,3′-pyrrolizine-1′,5′′-thia­zolidine]s containing four contiguous stereogenic centres have been synthesized with high regio- and stereoselectivity in a one-pot procedure using simple starting materials. The various modes of supra­molecular assembly depend upon different combinations of N—H⋯N, N—H⋯O, N—H⋯S=C and C—H⋯S=C hydrogen bonds.

## Introduction   

An attractive approach to the production of new organic com­pounds exhibiting broad-spectrum biological activity, for agricultural and pharmaceutical applications, is to combine in the same mol­ecule two or more pharmacophores of proven efficacy. We report here on the synthesis, characterization and structure of a new heterocyclic system containing three such units, namely, the spiro-oxindole, pyrrolizine and rhodanine (2-sulfanyl­idene­thia­zolidin-4-one) units. Spiro-oxindoles are an important class of alkaloids derived from indole that are widely distributed in nature, including examples such as elacomine [(2′*S*,3*R*)-6-hy­droxy-2′-(2-methyl­prop­yl)spiro­[1*H*-indole-3,3′-pyrrolidine]-2-one], horsfiline [(3*R*)-5-meth­oxy-1′-methyl­spiro­[1*H*-indole-3,3′-pyrrolidine]-2-one] rhynchophylline [methyl (7β,16*E*,20α)-16-(meth­oxy­methyl­ene)-2-oxocorynoxan-17-oate] and spiro­tryprostatin {(3*S*,3′*S*,5′a*S*,10′a*S*)-6-meth­oxy-3′-(2-methyl­prop-1-en­yl)spiro­[1*H*-indole-3,2′-3,5a,6,7,8,10a-hexa­hydro-1*H*-di­pyrrolo­[1,2-*c*:1′,4′-*f*]pyrazine]-2,5′,10′-trione}, and com­pounds of this type exhibit a wide range of biological activity, including anti­bacterial, anti­fungal, anti-oxidant and anti­tumour activity (Russel, 2010 ▸). In addition, pyrrolizines have been found to be promising scaffolds for anti­cancer drugs (Belal & El-Gendy, 2014 ▸), while com­pounds derived from rhodanine have been found to exhibit outstanding levels of anti­bacterial and anti­fungal activity (Sortino *et al.*, 2007 ▸; Tomasić & Masic, 2009 ▸). Hence, the synthesis of new com­pounds containing all three of these mol­ecular fragments, *i.e.* spiro-oxo­indole, pyrrolizine and rhodanine, is essential, and an efficient route to such com­pounds involves a 1,3-dipolar cyclo­addition reaction between an appropriate derivative of isatin (1*H*-indole-2,3-dione), an amino acid and an electron-deficient alkene (Ponnala *et al.*, 2006 ▸; Liu *et al.*, 2011 ▸).
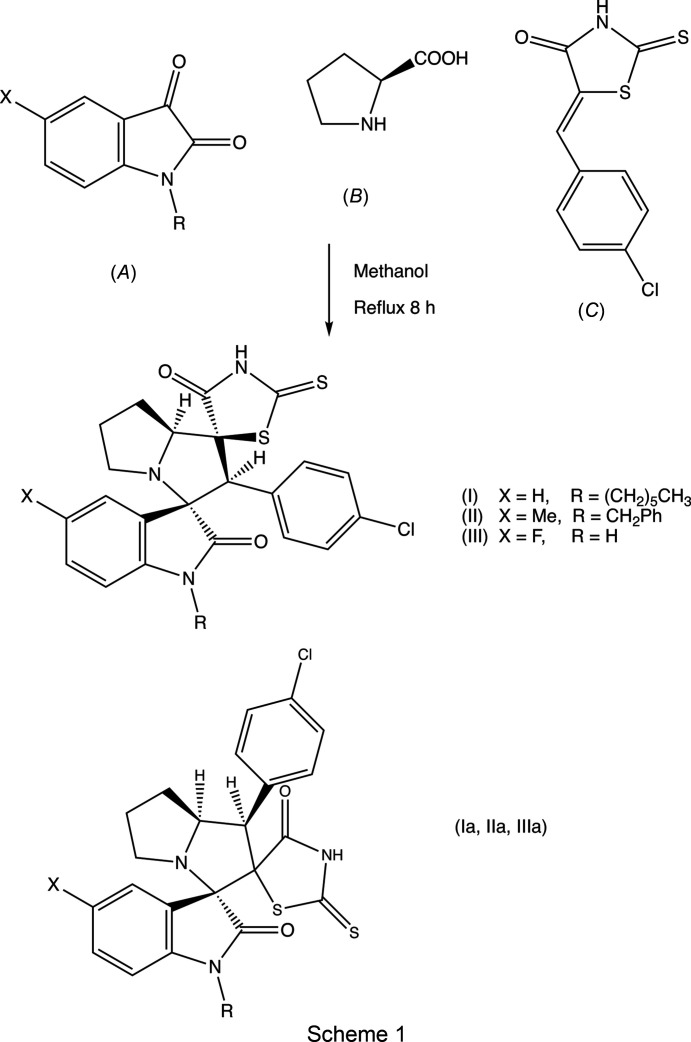


**Figure 1:**
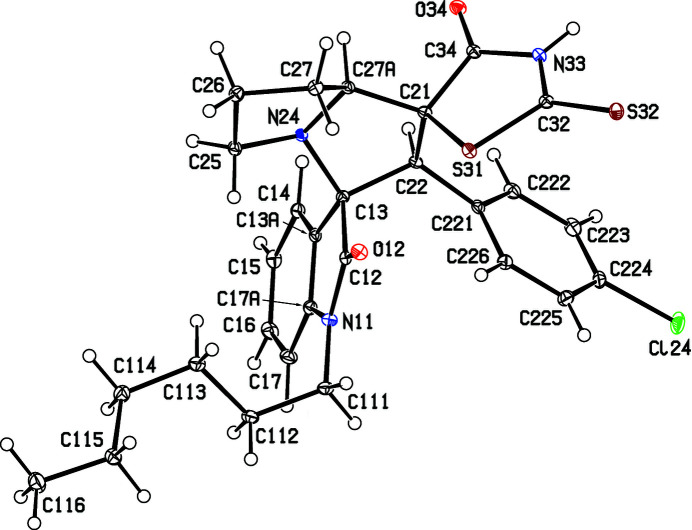
The mol­ecular structure of the (3*R*,1′*S*,2′*S*,7a′*S*) enanti­omer of com­pound (I), showing the atom-labelling scheme. Displacement ellipsoids are drawn at the 30% probability level.

Accordingly, we report here the synthesis and characterization of three examples, namely, (3*RS*,1′*SR*,2′*SR*,7a′*SR*)-2′-(4-chloro­phen­yl)-1-hexyl-2′′-sulfanyl­idene-5′,6′,7′,7a′-tetra­hydro-2′*H*-di­spiro­[indoline-3,3′-pyrrolizine-1′,5′′-thia­zolidine]-2,4′′-di­one, (I), (3*RS*,1′*SR*,2′*SR*,7a′*SR*)-2′-(4-chloro­phen­yl)-1-benzyl-5-methyl-2′′-sulfanyl­idene-5′,6′,7′,7a′-tetra­hydro-2′*H*-di­spiro­[in­doline-3,3′-pyrrolizine-1′,5′′-thia­zolidine]-2,4′′-dione, (II), and (3*RS*,1′*SR*,2′*SR*,7a′*SR*)-2′-(4-chloro­phen­yl)-5-fluoro-2′′-sul­fan­yl­idene-5′,6′,7′,7a′-tetra­hydro-2′*H*-di­spiro­[indoline-3,3′-pyrrolizine-1′,5′′-thia­zolidine]-2,4′′-dione, (III), along with the structures of com­pounds (I) and (II) (Figs. 1 ▸ and 2 ▸). The com­pounds were synthesized, as the sole isolated product in each case, in a one-pot procedure involving the reaction between an isatin, (*A*) [1-hexyl­isatin for (I), *N*-benzyl-5-methyl­isatin for (II) and 5-fluoro­isatin for (III)], l-proline, (*B*), and the electron-deficient alkene (*Z*)-5-(4-chloro­benzyl­idene)-2-sulfanyl­idene­thia­zolidin-4-one [5-(4-chloro­benzyl­idene)rhodanine], (*C*) (Scheme 1). Analysis of the NMR spectra showed which regioisomer had been isolated, while the crystal structure analyses for (I) and (II) established the relative stereochemistry at the four contiguous stereogenic centres at the atoms labelled here as C13, C21, C22 and C27*A* (Figs. 1 ▸ and 2 ▸), which correspond to the chemical sites C3, C1′, C2′ and C7a′, respectively, as defined in §2.3 below.

## Experimental   

### Synthesis and crystallization   

For the synthesis of com­pounds (I)–(III), a solution con­taining equimolar qu­anti­ties (0.39 mmol of each reactant) of l-proline, (*Z*)-5-(4-chloro­benzyl­idene)-2-sulfanyl­idene­thi­a­zoli­din-4-one and the appropriate isatin in methanol (120 ml) was heated under reflux for 8 h, after which time monitoring using thin-layer chromatography (TLC) indicated that the reactions were com­plete. Each solution was then allowed to cool to ambient temperature and the resulting solid product was collected by filtration. Crystals suitable for single-crystal X-ray diffraction were selected directly from the synthetic products.

### Analytical data   

#### Compound (I)   

Yield 52%, m.p. > 570 K. NMR (DMSO-*d*_6_): δ(^1^H) 0.76–0.90 (*m*, 3H), 1.10–1.32 (*m*, 6H), 1.40–1.54 (*m*, 2H), 1.75–1.89 (*m*, 1H), 1.89–1.99 (*m*, 1H), 1.99–2.13 (*m*, 2H), 2.41–2.48 (*m*, 1H), 2.52–2.57 (*m*, 1H), 3.51–3.69 (*m*, 2H), 4.42 (*t*, *J* = 7.22 Hz, 1H, H-7a′), 4.64 (*s*, 1H, H-2′), 6.86 (*d*, *J* = 7.61 Hz, 1H, H-7), 7.04 (*t*, *J* = 7.52 Hz, 1H, H-6), 7.14 (*d*, *J* = 8.59 Hz, 2H, H_*o*_), 7.18 (*t*, *J* = 7.71 Hz, 1H, H-5), 7.25 (*d*, *J* = 8.59 Hz, 2H, H_*m*_), 7.49 (*d*, *J* = 7.22 Hz, 1H, H-4), 13.21 (*br s*, 1H, NH-3′′); δ(^13^C) 14.3 (CH_3_), 22.5 (CH_2_), 26.6 (CH_2_), 27.4 (CH_2_), 28.1 (CH_2_), 30.0 (CH_2_), 31.3 (CH_2_), 40.0 (CH_2_), 47.3 (CH_2_), 65.3 (C-2′), 74.3 (C-spiro), 74.9 (C-7a′), 75.1 (C-spiro), 109.5 (C-7), 123.6 (C-6), 124.0 (C-4), 128.7 (C_*m*_), 129.0 (C), 129.9 (C-5), 130.5 (C), 132.4 (C_*o*_), 134.0 (C), 142.9 (C), 176.1 (N—C=O), 179.6 (N—C=O), 203.9 (C=S). MS–ESI (*m*/*z*) found for [*M* + H]^+^ 540.1543, C_28_H_31_ClN_3_O_2_S_2_ has an exact mass of 540.1546. MS–EI (70 eV) *m*/*z* (%) 499 (6), 284 [100, *M* ^+^ − 4-Cl-(C_6_H_4_)–CHC–CONHCS_2_], 255 {27, [4-Cl-(C_6_H_4_)–CHC–CONHCS_2_]}, 217 (17), 168 (82), 133 (33), 118 (25), 89 (38). Analysis (%) found: C 62.4, H 5.5, N 7.7; C_28_H_30_ClN_3_O_2_S_2_ requires: C 62.3, H 5.6, N 7.8.

#### Compound (II)   

Yield 50%, m.p. > 570 K. NMR (DMSO-*d*_6_): δ(^1^H) 1.84 (*dt*, *J* = 19.1, 17.4, 9.1 Hz, 1H, H-6′), 1.92–2.01 (*m*, 1H, H-7′), 2.02–2.14 (*m*, 2H, H-6′ & H-7′), 2.25 (*s*, 3H, 5-CH_3_), 2.54–2.61 (*m*, 1H, H-5′), 4.43 (*t*, *J* = 7.2 Hz, 1H, H-7a′), 4.67 (*s*, 1H, H-2′), 4.80 (*d*, *J* = 15.5 Hz, 1H, NC*H*H), 4.86 (*d*, *J* = 15.5 Hz, 1H, NCH*H*), 6.69 (*d*, *J* = 8.00 Hz, 1H), 6.93 (*d*, *J* = 7.61 Hz, 1H), 7.10 (*d*, *J* = 8.94 Hz, 2H), 7.14 (*d*, *J* = 8.76 Hz, 2H), 7.21 (*dd*, *J* = 7.55, 1.98 Hz, 2H), 7.27–7.37 (*m*, 4H), 13.22 (*br s*, 1H, H-3′′); δ(^13^C) 21.1 (5-CH_3_), 28.2 (CH_2_), 30.0 (CH_2_), 43.5 (N—CH_2_), 47.2 (CH_2_), 65.2 (C-2′), 74.4 (C-spiro), 74.9 (C-7a′), 75.2 (C-spiro), 109.8 (CH), 124.8 (CH), 128.0 (CH), 128.2 (CH), 128.8 (CH), 129.0 (C), 129.1 (CH), 130.1 (CH), 130.5 (C), 132.3 (CH), 133.1 (C), 133.9 (C), 136.3 (C), 140.1 (C), 176.2 (N—C=O), 179.6 (N—C=O), 203.8 (C=S). MS–EI (70 eV) *m*/*z* (%) 539 (5), 304 {60, *M* ^+^ − 4-Cl-(C_6_H_4_)–CHC–CONHCS_2_], 255 {4, [4-Cl-(C_6_H_4_)–CHC–CONHCS_2_]}, 236 (16), 213 (10), 168 (100), 133 (40), 123 (8), 91 [75, (C_7_H_7_)^+^], 89 (55). Analysis (%) found: C 64.4, H 4.6, N 7.4; C_30_H_26_ClN_3_O_2_S_2_ requires: C 64.3, H 4.7, N 7.5.

#### Compound (III)   

Yield 49%, m.p. > 570 K. NMR (DMSO-*d*_6_): δ(^1^H) 1.76–1.89 (*m*, 1H, H-6′), 1.89–1.99 (*m*, 1H, H-7′), 2.00–2.12 (*m*, 2H, H-6′ & H-7′), 2.53–2.64 (*m*, 1H, H-5′), 4.41 (*t*, *J* = 7.3 Hz, 1H, H-7a′), 4.66 (*s*, 1H, H-2′), 6.62 (*dd*, *J* = 8.4, 4.3 Hz, 1H), 6.92 (*ddd*, *J* = 11.1, 8.7, 2.7 Hz, 1H), 7.24 (*d*, *J* = 8.6, Hz, 2H, H_*o*_), 7.31 (*d*, *J* = 8.6 Hz, 2H, H_*m*_), 7.37 (*dd*, *J* = 8.1, 2.7 Hz, 1H), 10.73 (*s*, 1H, NH), 13.19 (*br s*, 1H, NH-1), 13.19 (*br s*, 1H, H-3′′); δ(^13^C) 28.2 (CH_2_), 29.9 (CH_2_), 47.3 (CH_2_), 65.0 (C-2′), 74.3 (C-spiro), 75.0 (C-7a′), 75.9 (C-spiro), 111.2 (*d*, *J*_CF_ = 7.3 Hz, C), 112.2 (*d*, *J*_CF_ = 24.8 Hz, CH), 116.2 (*d*, *J*_CF_ = 23.3 Hz, CH), 128.9 (CH), 130.6 (C), 131.7 (*d*, *J*_CF_ = 7.3 Hz, C—N), 132.3 (CH), 134.1 (C), 137.9 (C), 157.8 (C), 159.0 (*d*, *J*_CF_ = 237 Hz, C—F), 160.2 (C), 178.3 (N—C=O), 179.5 (N—C=O), 204.0 (C=S). MS–EI (70 eV) *m*/*z* (%) 473 (1.3, *M* ^+^), 368 (3), 355 (6), 255 [6, 4-Cl-(C_6_H_4_)-CHC-CONHCS_2_], 218 {20, *M* ^+^ − [4-Cl-(C_6_H_4_)–CHC–CONHCS_2_]}, 215 (51), 168 (17), 160 (20), 127 (35), 111 (18), 97 (37), 81 (49), 55 (75), 44 (100). Analysis (%) found: C 55.7, H 3.7, N 8.8; C_22_H_17_ClFN_3_O_2_S_2_ requires: C 55.8, H 3.6, N 8.9.

### Refinement   

Crystal data, data collection and structure refinement details are summarized in Table 1 ▸. The atom labelling for the central di­spiro unit is based on the systematic chemical numbering, following the convention used previously (Quiroga *et al.*, 2017 ▸); thus, the atoms with chemical locants N1, C2 and so on are labelled here as N11, C12, *etc.*; those atoms with chemical locants such as C1′, C2′ and so on are labelled here as C21, C22, *etc*.; and those atoms with chemical locants such as S1′′, C2′′ and so on are labelled here as S31, C32, *etc*. All other chemical fragments are treated as substituents on the central di­spiro unit. All H atoms were located in difference maps. H atoms bonded to C atoms were subsequently treated as riding atoms in geometrically idealized positions, with C—H = 0.95 (aromatic), 0.98 (CH_3_), 0.99 (CH_2_) or 1.00 Å (aliphatic C—H), and with *U*_iso_(H) = *kU*_eq_(C), where *k* = 1.5 for the methyl groups, which were permitted to rotate but not to tilt, and 1.2 for all other H atoms bonded to C atoms. For the H atoms bonded to N atoms, the atomic coordinates were refined with *U*_iso_(H) = 1.2*U*_eq_(N), giving an N—H distance of 0.862 (17) Å in (I) and 0.77 (3) Å in (II). For com­pound (II), the correct orientation of the structure with respect to the polar-axis direction was calculated by means of the Flack *x* parameter (Flack, 1983 ▸), with *x* = 0.050 (19) as calculated (Parsons *et al.*, 2013 ▸) using 2956 quotients of the type [(*I* ^+^) − (*I* ^−^)]/[(*I* ^+^) + (*I* ^−^)].

## Results and discussion   

Compounds (I)–(III) (Scheme 1) were each isolated as a single stereoisomer in yields of 53% for (I), 49% for (II) and 50% for (III). For all three products, the com­positions were established by elemental analysis, com­plemented by high-resolution mass spectrometry in the case of (I) (§2.1). The ^1^H and ^13^C NMR spectra contained all the signals expected for the proposed formulations, and the regioselectivity of the reactions leading to the products was established by analysis of the ^1^H spectra; it is necessary here to discuss only the analysis for (I), as those for (II) and (III) follow entirely similar lines. For (I), the signal from the proton H2′ bonded to atom C2′ (atom C22 in the crystallographic labeling scheme; see Fig. 1 ▸ and §2.2) was ob­served as a singlet at δ 4.64, while the signal for H7a′ bonded to C7a′ (C27*A*) was observed as a triplet (*J* = 7.22 Hz) at δ 4.42. These two signals indicate the formation of the pyrrolizine in (I), singly substituted at position C2′ and doubly substituted at positions C1′ and C3′, so confirming the identity of regioisomer (I) (Scheme 1 and Fig. 1 ▸). Had the alternative regioisomer (I*a*) been formed, the appearance of these two pyrrolizine signals would have been different; that for atom H7a′ would have been a doublet of triplets and, crucially, that for atom H1′ would have appeared as a doublet, rather than the singlet actually observed. Entirely similar remarks apply to the spectra of com­pounds (II) and (III) but, in addition, five of the signals in the ^13^C NMR spectrum of (III) exhibit coupling to the ^19^F nucleus at position 5, namely, those at δ 159.0 for C5, 131.7 for C7, 116.2 and 112.2 for C4 and C6, and 111.2 for C3*A*; the four-bond coupling to atom C7*A* is too small to be resolved.

Although the regiochemistry of the synthetic reactions can be deduced from the NMR data, it is not possible to establish from these data the relative stereochemistry of all four stereogenic centres, but this is readily achieved by crystal structure analysis. The space groups for com­pounds (I) and (II) (Table 1 ▸) show that they have both crystallized as racemic mixtures and, for each com­pound, the reference mol­ecule was selected as that having the *R* configuration at atom C13 (Figs. 1 ▸ and 2 ▸); on this basis, the configuration at each of atoms C21, C22 and C27*A* is *S*, with these atoms corresponding, respectively, to locants C3, C1′, C2′ and C7a′ in the chemical numbering scheme, so that the overall configuration of these com­pounds is (3*RS*,1′*SR*,2′*SR*,3′*SR*).

Based on earlier work (Pardasani *et al.*, 2003 ▸; Quiroga *et al.*, 2017 ▸), a reaction sequence can be proposed which commences with nucleophilic addition of the proline com­ponent (*B*) to the isatin (*A*) (Scheme 1) to form the inter­mediate (*D*) (Scheme 2), followed by sequential cyclo­dehydration to give (*E*) and deca­rboxylation to form the key azomethine inter­mediate (*F*). This inter­mediate then undergoes a 1,3-dipolar cyclo­addition with the electron-deficient alkene (*C*) to form the products (I)–(III). The alternative orientation of the alkene relative to the azomethine in the addition reaction would give the products (I*a*)–(III*a*) with transposed chloro­phenyl and rhodanine units, but these have not been detected. Thus, the negative pole of inter­mediate (*F*) has coupled to the heterocyclic end of the alkenic double bond, adjacent to the carbonyl group, rather than to the chloro­phenyl end. Neither of the com­ponents in the cyclo­addition reaction step contains any stereogenic centres, and there are no reagents present which could induce enanti­oselectivity; hence the products are formed as racemic mixtures. These each contain four contiguous stereogenic centres, so that whichever of these centres is formed first, it appears to exert strong control over the formation of all the others. In the transition state leading to the formation of the products (I)–(III), the reactants can approach one another in two orientations: the *endo* transition state, in which the Cl atom is remote from the aryl ring of the isatin unit, leads to the observed (3*RS*,1′*SR*,2′*SR*,3′*SR*) stereochemistry, whereas the alternative *exo* transition state, with the Cl atom close to the aryl ring of the isatin, would lead to the alternative (3*RS*,1′*RS*,2′*RS*,3′*RS*) stereochemistry, which is not observed. The choice of the transition state in this step is presumably determined by the minimization of steric hindrance. Hence this proposed reaction mechanism can account for both the regiochemistry and for the relative stereochemistry at the four stereogenic centres.
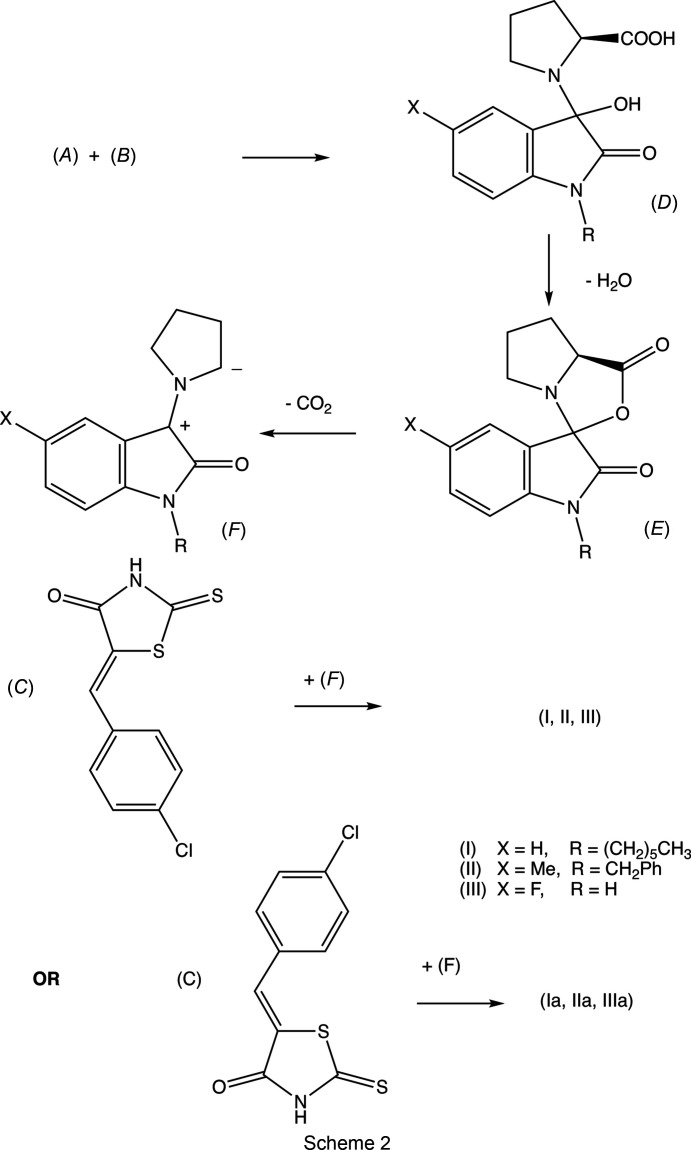


Within the mol­ecules of (I) and (II), the rhodanine rings are almost planar, with r.m.s. deviations from the mean planes of the five ring atoms of 0.0573 Å in (I) and 0.0210 Å in (II). However, the rings containing atoms C22 and C25 (Figs. 1 ▸ and 2 ▸) both adopt half-chair conformations, as indicated by the ring-puckering parameters (Cremer & Pople, 1975 ▸) shown in Table 2 ▸. For an idealized half-chair conformation, the value of φ_2_ is (36*k* + 18)°, where *k* represents an integer (Boeyens, 1978 ▸). Here the rings denoted *A* (Table 2 ▸) are twisted about a line joining atom C27*A* to the mid-point of the C13—C22 bond, while the rings denoted *B* are twisted about a line joining atom N24 to the mid-point of the C26—C27 bond.

Overall, therefore, the com­position of com­pounds (I)–(III) has been determined; the constitutions, including the regiochemistry, have been established from the NMR spectra and the relative configurations of the stereogenic centres, and the conformations of the nonplanar rings in (I) and (II) have been established from the X-ray structure analyses. We have also investigated the crystal structure of com­pound (III). Despite repeated attempts at crystallization, this com­pound consistently formed tightly-packed clusters of very thin lath-like crystals, and the resulting diffraction data and the structure deduced from it is of somewhat indifferent quality (see supporting information). After conventional refinement of (III), the resulting difference map contained several significant electron-density maxima, but no chemically plausible solvent model could be developed from these peaks. Accordingly, SQUEEZE (Spek, 2015 ▸) was applied and the refinement using this modified data set established that the constitution and configuration of (III) are the same as those for com­pounds (I) and (II), and that the conformations of the type *A* and *B* rings are also very similar to those in (I) and (II) (Table 2 ▸). However, the identity of the included solvent species remains undetermined.

**Figure 3:**
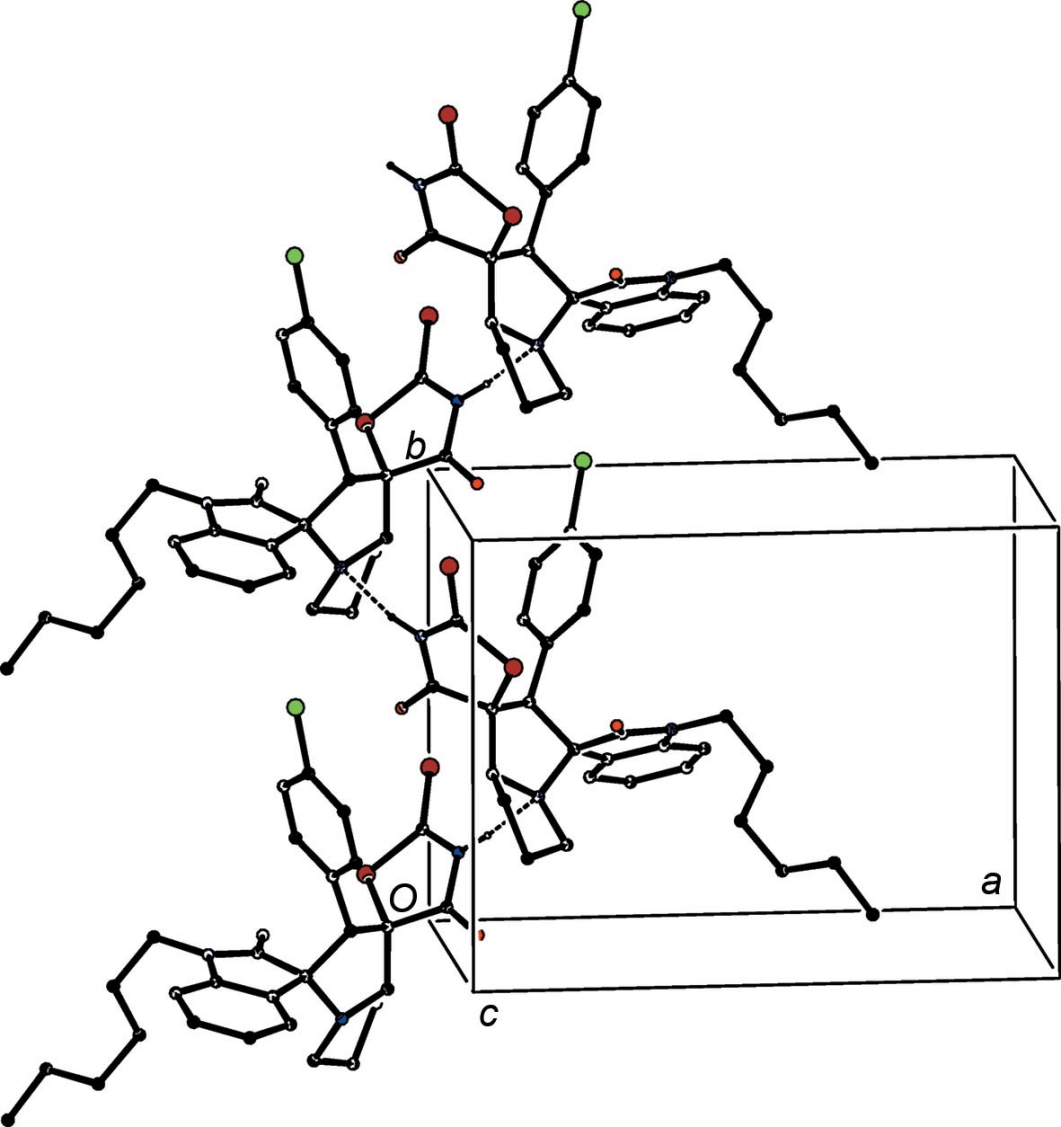
Part of the crystal structure of com­pound (I), showing the formation of a hydrogen-bonded *C*(6) chain running parallel to [010]. Hydrogen bonds are drawn as dashed lines and, for the sake of clarity, H atoms bonded to C atoms have all been omitted.

**Figure 4:**
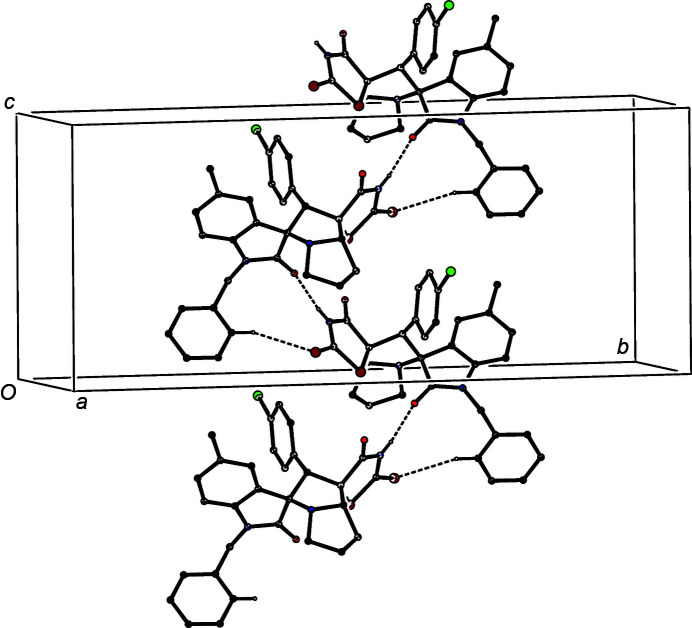
Part of the crystal structure of com­pound (II), showing the formation of a hydrogen-bonded *C*(8)*C*(12)[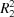
(11)] chain of rings running parallel to [010]. Hydrogen bonds are drawn as dashed lines and, for the sake of clarity, H atoms bonded to C atoms have all been omitted.

**Figure 5:**
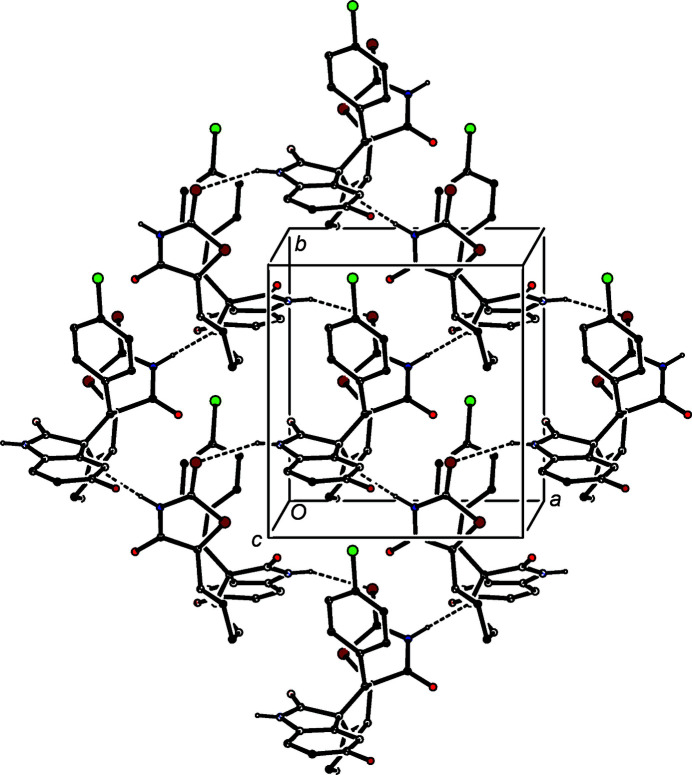
Part of the crystal structure of com­pound (III), showing the formation of a hydrogen-bonded sheet of 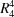
(24) rings; see supporting information for full details. Hydrogen bonds are drawn as dashed line lines and, for the sake of clarity, H atoms bonded to C atoms have all been omitted.

The supra­molecular assembly of both (I) and (II) is very simple. In com­pound (I), a single N—H⋯N hydrogen bond (Table 3 ▸) links mol­ecules which are related by a 2_1_ screw axis to form simple *C*(6) chains (Etter, 1990 ▸; Etter *et al.*, 1990 ▸; Bernstein *et al.*, 1995 ▸) running parallel to the [010] direction (Fig. 3 ▸), but there are no direction-specific inter­actions between adjacent chains. In com­pound (II), mol­ecules which are related by a 2_1_ screw axis are linked by a combination of one N—H⋯O hydrogen bond and one C—H⋯S=C hydrogen bond (Table 3 ▸). These two inter­actions, acting singly, give rise to *C*(8) and *C*(12) chains, respectively, while in combination they generate a *C*(8)*C*(12)[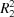
(11)] chain of rings (Fig. 4 ▸). There are no direction-specific inter­actions between adjacent chains. The crystal structure of (III) contains N—H⋯N and N—H⋯S hydrogen bonds (Table 3 ▸), which individually generate *C*(6) and *C*(9) chains, both running parallel to the [010] direction, and in combination these inter­actions generate a sheet of 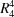
(24) rings lying parallel to (001) (Fig. 5 ▸).

A number of structures have been reported for spiro­[in­doline-3,3′-pyrrolizine] derivatives (Sarrafi & Alimohammadi, 2008*a* ▸,*b* ▸; Sathya *et al.*, 2012 ▸; Fathimunnisa *et al.*, 2015 ▸; Corres *et al.*, 2016 ▸), but often without any mention of either the relative or the absolute stereochemistry, despite the presence of multiple stereogenic centres.

## Supplementary Material

Crystal structure: contains datablock(s) global, I, II. DOI: 10.1107/S2053229620009791/ky3198sup1.cif

Structure factors: contains datablock(s) I. DOI: 10.1107/S2053229620009791/ky3198Isup2.hkl

Structure factors: contains datablock(s) II. DOI: 10.1107/S2053229620009791/ky3198IIsup3.hkl

Crystal structure: contains datablock(s) III. DOI: 10.1107/S2053229620009791/ky3198IIIsup4.cif

CCDC references: 2017035, 2017034

## Figures and Tables

**Figure 2:**
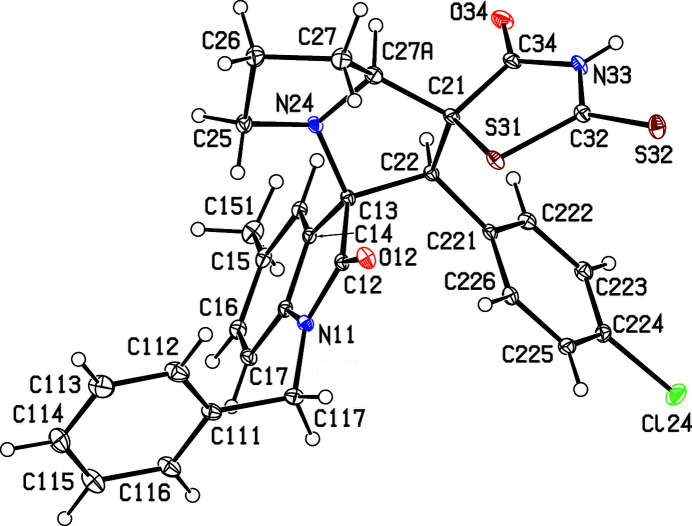
The mol­ecular structure of the (3*R*,1′*S*,2′*S*,7a′*S*) enanti­omer of com­pound (II), showing the atom-labelling scheme. Displacement ellipsoids are drawn at the 30% probability level.

**Table 1:** Experimental details For both structures: *Z* = 4. Experiments were carried out at 100 K with Mo *K*α radiation using a Bruker D8 Venture diffractometer. Absorption was corrected for by multi-scan methods (*SADABS*; Bruker, 2016 ▸). H atoms were treated by a mixture of independent and constrained refinement.

	(I)	(II)
Crystal data		
Chemical formula	C_28_H_30_ClN_3_O_2_S_2_	C_30_H_26_ClN_3_O_2_S_2_
*M*_r_	540.12	560.11
Crystal system, space group	Monoclinic, *P*2_1_/*c*	Orthorhombic, *P* *n* *a*2_1_
*a*, *b*, *c* (Å)	14.1669 (5), 10.7145 (3), 17.1350 (5)	8.2419 (2), 28.0429 (6), 11.5843 (3)
α, β, γ (°)	90, 98.654 (1), 90	90, 90, 90
*V* (Å^3^)	2571.33 (14)	2677.44 (11)
μ (mm^−1^)	0.34	0.33
Crystal size (mm)	0.18 × 0.11 × 0.05	0.34 × 0.18 × 0.12

Data collection		
*T*_min_, *T*_max_	0.906, 0.983	0.917, 0.961
No. of measured, independent and observed [*I* > 2σ(*I*)] reflections	58781, 6390, 5580	26105, 6574, 6425
*R*_int_	0.049	0.038
(sin θ/λ)_max_ (Å^−1^)	0.667	0.667

Refinement		
*R*[*F* ^2^ > 2σ(*F* ^2^)], *wR*(*F* ^2^), *S*	0.032, 0.075, 1.03	0.026, 0.064, 1.05
No. of reflections	6390	6574
No. of parameters	329	347
No. of restraints	0	1
Δρ_max_, Δρ_min_ (e Å^−3^)	0.47, −0.41	0.37, −0.27
Absolute structure	–	Flack *x* determined using 2956 quotients [(*I* ^+^) − (*I* ^−^)]/[(*I* ^+^) + (*I* ^−^)] (Parsons *et al.*, 2013 ▸)
Absolute structure parameter	–	0.050 (19)

Computer programs: *APEX3* (Bruker, 2018 ▸), *SAINT* (Bruker, 2017 ▸), *SHELXT2014* (Sheldrick, 2015*a* ▸), *SHELXL2014* (Sheldrick, 2015*b* ▸) and *PLATON* (Spek, 2020 ▸).

**Table 2:** Ring-puckering parameters (Å, °) Parameters for rings *A* and *B* are calculated for the atom sequences N24–C13–C22–C21–C27*A* and N24–C25–C26–C27–C27*A*, respectively. Data for (III) are available in the supporting information.

Ring *A*		(I)	(II)	(III)
*Q*_2_		0.4311 (13)	0.411 (2)	0.424 (6)
φ_2_		55.57 (17)	61.9 (3)	54.2 (8)
				
Ring *B*		(I)	(II)	(III)
*Q*_2_		0.4103 (14)	0.416 (3)	0.390 (7)
φ_2_		270.63 (18)	269.1 (3)	272.3 (9)

**Table 3:** Hydrogen-bond parameters (Å, °) The data for (III) are available in the supporting information.

	*D*—H⋯*A*		*D*—H	H⋯*A*	*D*⋯*A*	*D*—H⋯*A*
(I)	N33—H33⋯N24^i^		0.863 (17)	2.079 (17)	2.8933 (15)	157.2 (16)
(II)	N33—H33⋯O12^ii^		0.77 (3)	2.05 (3)	2.811 (2)	170 (3)
	C112—H112⋯S32^iii^		0.95	2.88	3.760 (3)	155
(III)	N11—H11⋯S32^iv^		0.88	2.55	3.374 (5)	156
	N33—H33⋯N24^v^		0.88	2.08	2.878 (7)	150

Symmetry codes: (i) −*x*, *y* + 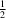, −*z* + 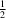; (ii) −*x* + 1, −*y* + 1, *z* + 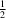; (iii) −*x* + 1, −*y* + 1, *z* − 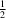; (iv) −*x*, *y* − 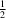, −*z* + 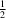; (v) −*x* + 1, *y* + 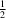, −*z* + 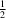.
